# Measurement Strategies for the Classification of Edible Oils Using Low-Cost Miniaturised Portable NIR Instruments

**DOI:** 10.3390/foods10112856

**Published:** 2021-11-18

**Authors:** Barbara Giussani, Alix Tatiana Escalante-Quiceno, Ricard Boqué, Jordi Riu

**Affiliations:** 1 Dipartimento di Scienza e Alta Tecnologia, Università degli Studi dell’Insubria, Via Valleggio, 9, 22100 Como, Italy; barbara.giussani@uninsubria.it; 2 Department of Analytical Chemistry and Organic Chemistry, Universitat Rovira i Virgili, Carrer Marcel·lí Domingo 1, 43007 Tarragona, Spain; aescalantequiceno@gmail.com (A.T.E.-Q.); ricard.boque@urv.cat (R.B.)

**Keywords:** olive oil, miniaturised instrumentation, measurement strategies, near-infrared spectroscopy, classification

## Abstract

Miniaturised near-infrared (NIR) instruments have been increasingly used in the last few years, and they have become useful tools for many applications on different types of samples. The market already offers a wide variety of these instruments, each one having specific requirements for the correct acquisition of the instrumental signal. This paper presents the development and optimisation of different measuring strategies for two miniaturised NIR instruments in order to find the best measuring conditions for the rapid and low-cost analysis of olive oils. The developed strategies have been applied to the classification of different samples of olive oils, obtaining good results in all cases.

## 1. Introduction

Olive oil is the natural oil extracted from olives (the fruit of olive trees), typically containing 14% saturated fat, 73% monounsaturated fat (mostly oleic acid), a modest amount of vitamin E (13% of the daily value) and vitamin K (7% of the daily value). Extra virgin olive oil also contains antioxidants that have powerful health benefits [1]. Olive oil is commonly used in cooking (especially in the Mediterranean basin), but it is also used in cosmetics and soaps, in pharmaceuticals, as a fuel and lubricant, and has a use in certain religious rites. Spain is the largest producer of olive oil [2] with 1387 thousand tonnes in 2020/21 according to the olive oil sector council of Spanish Cooperativas Agro-Alimentarias (agri-food cooperatives) [3].

The International Olive Council (IOC) classifies olive oils suitable for human consumption—extra virgin olive oil (EVOO), virgin olive oil (VOO), refined olive oil (ROO) and pomace olive oil (POO)—and lampante oil, which is intended for refining or for technical uses [4,5]. Of these four grades suitable for human consumption, EVOO is considered the highest quality grade. The IOC also establishes some guidelines to control the quality, purity and authenticity of olive oils [6] and invites experts in chemistry to collaborate in developing new analytical methods considering the technical advances in instrumentation capable of accurately and reliably analysing olive oil.

Among the analytical techniques that can be used to characterise olive oil samples, chromatographic techniques (liquid and gas chromatography) and infrared spectroscopy are the most widely applied, although other techniques such as fluorescence spectroscopy, Raman spectroscopy, ultraviolet and visible spectroscopy or nuclear magnetic resonance spectroscopy, have also been used [7]. Of all these techniques, near infrared (NIR) spectroscopy has proved to be one of the most effective, as described in the review that was recently written by Li and co-authors [8]. This review deals with NIR spectroscopy methods for non-destructive quality analysis of edible oils, being benchtop NIR instrumentation, a well-known and consolidated technique, the most used method in this field. Portable and miniaturised NIR spectrometers have been successfully employed in olive oil research, even if the reported applications are still few. There is not a clear consensus in the literature about the nomenclature and the distinction between portable and miniaturised NIR devices [9], but miniaturised instruments seem to have implemented new technological solutions, improving light sources, wavelength selection techniques (including techniques based on micro-electromechanical systems (MEMS) or micro-opto-electromechanical systems (MOEMS)) and detectors. Focusing on the size, field spectrometers (in contrast to benchtop instruments) may be divided into transportable (deployable on field while mounted in a car), portable in a suitcase (>4 kg of total weight) and handheld (<1 kg) ones [10]. Miniaturised instruments would be those comparable (or less) to the size of a book. The advantages of miniaturised NIR sensors lie in the possibility of analysing the samples directly on the field, often without any sample pre-treatment or any transportation costs and with minimal waste and time of analysis. These sensors have a reduced cost if compared with benchtop NIR spectrometers; if a typical laboratory NIR instrument costs around EUR 30–40,000, miniaturised NIR instruments can be found for a few thousand euros, meaning a decrease of at least one order of magnitude in the price, and they could be potentially used also by non-scientists when the sensor is calibrated for the required application: chemometric modelling is mandatory to extract the required information from the spectroscopic signal.

Several NIR sensors can be found on the market, with different spectroscopic and technical characteristics and costs [9]. Viavi Solution Inc. (previously named JDSU) offers sensors equipped with a transmission accessory that allows the analysis of liquid samples in an effective and simple way. Yan et al. [11] described a method able to classify EVOO samples from other olive oil grades by their spectral data through a one-class classification approach, using an NIR Pro 1700 ES in the transmission mode. In this paper, the EVOO class mainly overlapped with the ROO class and less with the POO class, but a quite good classification was achieved. Borghi et al. [12] employed a MicroNIR Pro 1700 using two accessories for transmission and reflectance modes to qualitatively and quantitatively estimate the content of vegetable oils in the samples. The authors concluded that the transmission mode was the most efficient in terms of the limit of detection (LOD). Karunathilaka et al. [13] employed a microPHAZIR NIR device (Thermo Fisher Scientific, Waltham, MA, USA) in the transflection mode for authenticity purposes: they analysed 88 commercial samples labelled as EVOO but only 44% of the samples were classified as authentic EVOO (they used 24 authentic EVOO samples to build the class). In the conclusions, the authors stated that the misclassification could be attributed to the limited number of authentic EVOO samples, which were not representative of all the commercial samples investigated, but the hypothesis of the overlapped classes should not be discarded. Transflection was also employed by Weesepoel et al. [14], who developed a prototype sensor coupling NIR, UV-Vis and a miniaturised RGB-camera. Using all the available data simultaneously (in a high-level data fusion approach) and a one-class classification approach, they were able to successfully detect the adulteration of EVOO.

These papers are interesting and definitely pave the way for future studies on olive oil. However, they employ miniaturised instruments whose cost is not negligible and of which extra accessories are also commercially available. Our goal is to prove the suitability of much cheaper sensors, such as SCiO (Consumer Physics, Herzliya, Israel) and NeoSpectra Micro Development kit (Si-Ware Systems, Cairo, Egypt), in the classification of olive oil samples.

The SCiO and NeoSpectra Micro Development kits are quite different from each other in the way they measure the sample and in the spectroscopic range they use. SCiO covers the spectroscopic range between 740 and 1070 nm, including a part of the visible spectrum: for this reason, the colour of the sample may affect the measurements. It can be used in contact with the sample (if solid) or at a distance. The NeoSpectra Micro Development kit allows analysis between 1350 and 2558 nm and needs to be used in contact with the sample. Moreover, it has a quite large surface (2 × 2 cm) that must be fully covered by the sample during the analysis, as specified by the producer. The analysis of liquid samples is not obvious in this case.

These two sensors have also been successfully employed in milk analysis [15] with the aim to predict fat and protein contents. In the case of the NeoSpectra Micro Development kit, a quartz cuvette was used as the sample holder in reflection mode. For olive oil samples, we did not choose the same analytical strategy to perform the measurements. Olive oil is a viscous and fatty liquid sample, and the cuvette washing (the process that needs chemicals and generates wastes) and the drying time are certainly obstacles to the development of a simple and truly portable analytical method. The use of disposable optical glass vials proved to be a good alternative to quartz cuvettes when used in olive oil analysis with a benchtop NIR instrument in transmission mode [16] and may overcome the problem of cuvette washing. Moreover, learning from the results obtained in the milk analysis, the reflection mode of acquisition in direct liquid samples using the NeoSpectra Micro Development kit was avoided. In milk analysis, the information contained in the spectra was strongly related to the physical properties of the sample. This was an advantage because the physical properties of the milk reflected the content of macronutrients (especially the fat content). However, olive oil samples do not provide distinct visual differences regarding quality properties [17], and a quite more elaborate analytical chemistry strategy is thus needed. For this reason, the transflection mode of acquisition was also considered.

In this article, different analytical strategies for the analysis of olive oil samples with the SCiO and the NeoSpectra Micro Development kit miniaturised NIR spectrometers are presented. The attention is focused on the sample measurement method, optimising the required instrumental parameters, in an effort to make it fast, efficient, affordable and accessible from the economical point of view. The aim of the work was to develop analytical protocols of measurement suitable for different types of olive oils and being able to provide the best classification between them, showing that these protocols can also be applied to other types of edible oils, regardless of their physical characteristics (e.g., olive oil and sunflower oil have different colours or viscosity) and to differentiate between olive oils and seed oils. As a proof of concept, sunflower oil was selected to differentiate it from olive oil. Linear discriminant analysis (LDA) and partial least squares discriminant analysis (PLS-DA) were used for the classification, and we obtained good results with all the measurement strategies tested and the two miniaturised instruments.

## 2. Materials and Methods

### 2.1. Instrumentation

SCiO v1.2 (Consumer Physics, Herzliya, Israel) and NeoSpectra Micro Development Kit (Si-Ware Systems, Cairo, Egypt) were the two miniaturised instruments used for the analysis of the different oils. While SCiO can perform either contact or distance measurements, the main applications of the NeoSpectra Micro Development Kit are the analysis of solid samples that are in contact with the optical window. Both instruments work in reflectance mode. SCiO is a miniaturised NIR device using a molecular sensor with dimensions of 67.7 × 40.2 × 18.8 mm and a weight of 35 g. The wavelength range of SCiO is 740 to 1070 nm. The default scan time is between 2–5 s, and it cannot be set manually. Calibration of the instrument must be performed each time the instrument is switched on using the SCiO supplied reflectance standard, which is located inside the SCiO cover. The device is connected via Bluetooth to smartphones (Android and IOS) using the app ‘SCiO Lab’, and the recorded data is stored in the cloud. For data collection, the optical head of the device must be positioned touching the sample or at a distance of less than 1 cm. The illumination window and the detector are in the optical head. The light source illuminates the sample, and the detector captures the reflected light.

The NeoSpectra Micro Development Kit consists of a monolithic microelectromechanical system (MEMS) Michelson interferometer and a single InGaAs photodetector. The dimensions of the device are 32 × 32 × 22 mm with a weight of 17 g. The wavelength range of the NeoSpectra Micro Development Kit is 1350 to 2558 nm with a resolution of 16 nm. The scan time can be set manually. Calibration of the instrument must be performed when the instrument is switched on using a reflectance standard. A Spectralon (99% reflectance) reflectance standard was used. The device is connected to a PC via a universal serial bus (USB). The software package (SpectroMOST) allows monitoring the measured spectra, as well as configuring some parameters such as the scan time and the display mode (reflectance or absorbance). To perform the measurements, an initial warm-up time (30–40 min was used) is required, and the samples must be in contact with the optical window of the instrument. The optical window (2 × 2 cm) contains the detector and the light source, which consists of three tungsten halogen lamps. The spectra are stored in the connected PC.

### 2.2. Samples

A total of 66 samples of commercial oils were purchased in supermarkets in Tarragona, Spain, and stored at room temperature in a dry and dark place. Thirty-two samples were extra virgin olive oils (EVOO), 16 refined olive oils (ROO), 4 virgin olive oils (VOO) and 4 pomace olive oils (POO). Additionally, 10 refined sunflower oils (SFO) were also purchased in order to check if the optimised measuring methods worked for other types of oils (different, for instance, in colour and viscosity) than olive oils. The quality of the oils was obtained from the commercial oil labels. The percentages of the different oils try to mimic the percentage of oils that can be found in a typical Spanish supermarket.

For the external validation, the training set was made of 38 olive oil samples and 6 sunflower oil samples, and the test set was made of 18 olive oil samples and 4 sunflower oil samples. Both training and test sets were randomly split before the instrumental analysis and calculations. The different classes of the oils were representatively varied in the training and test sets. The same samples in the training and test sets were used in all the models and calculations.

### 2.3. Materials and Instruments

Disposable borosilicate glass vials of 22 × 38 mm (Ø × H) with a flat bottom design (DWK Life Sciences, Mainz, Germany) were used to contain the edible oils in the SCiO measurements. Qualitative filter paper grade 304 ClearLine (DD Biolab, Barcelona, Spain) hand-cut to a size of 20 × 20 mm was used to collect the sample and perform the analysis with NeoSpectra. Cover slips of 22 × 22 mm of borosilicate glass of hydrolytic class 1 with a thickness of 0.13–0.17 mm (Knittel Glass, Bielefield, Germany) were used to cover the sample in the analysis with NeoSpectra. Rings of poly(acrylonitrile-co-butadiene) of 20 × 2 mm (Ø × W) in black colour (JIOrings, Galdakao, Spain) were used to contain the sample in the analysis with NeoSpectra. Loctite Super Glue-3 brush, a cyanoacrylate-based adhesive of transparent colour with a fixing time between 15–60 s (Henkel Ibérica, SA, Barcelona, Spain), was used to fix the rings of poly(acrylonitrile-co-butadiene) to the cover slips in the analysis with NeoSpectra. MakerBot Replicator^®^ 2 Desktop 3D Printer (MakerBot Industries, New York, USA) was used to fabricate the holder used in the analysis with NeoSpectra. Quartz cuvettes model 101-20-40 (45 × 12.5 × 22.5 mm) with an optical path of 10 mm and model 404-1-46 (47.5 × 3.5 × 23.6 mm) with an optical path of 1 mm (Hellma, Jena, Germany) were used in the analysis with NeoSpectra.

### 2.4. Statistical Data Analysis

PLS Toolbox 8.9.1 (Eigenvector Inc., Manson, WA, USA) for Matlab 2021a (Mathworks Inc., Natick, MA, USA) and Matlab with the Statistics and Machine Learning Toolbox were used for data analysis. Linear Discriminant Analysis (LDA) was performed with Matlab and the Statistics and Machine Learning Toolbox, and Principal Component Analysis (PCA) and Partial Least Squares Discriminant Analysis (PLS-DA) were performed with the PLS Toolbox. Different spectral pre-processing methods were tested: multiplicative scatter correction (MSC), standard normal variate (SNV) and first and second Savitzky–Golay derivatives with a different number of smoothing points (from 7 to 21 points). After spectral pre-processing, data were finally mean-centred. In the case of PLS-DA, the Venetian blinds method (with 23 data splits and 5 samples per blind) was used for cross-validation. Sensitivity, specificity, precision, accuracy, negative predictive value (NPV), false positive rate (FPR) and false negative rate (FNR) were the parameters used to assess the goodness of the classification [18].

## 3. Results and Discussion

### 3.1. Optimisation of the Instrumental Setup

Different procedures were developed to optimise the measurement procedures with the aim of obtaining the best analytical signals and the best classification results.

#### 3.1.1. SCiO

Initially, measurements were made by placing an oil drop (different volumes from 25 to 200 μL were tested) on a borosilicate cover slip covered with an aluminium foil on the top of it to enhance reflectance and measuring from the opposite side, but no spectral information was obtained from the oil in this way. In a further stage, different volumes of oil were also deposited in glass vials, and measurements were taken from the base and from the nozzle of the vial. From the nozzle of the vial, the signal was more intense but less stable than from the base of the vial.

**Figure 1:**
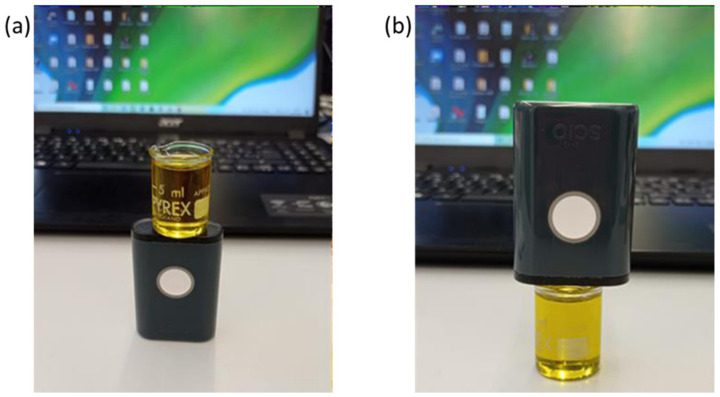
Pictures of the SCiO experimental set-up measuring from (**a**) the base and (**b**) the nozzle of the vial.

Finally, to contain the oil samples during the SCiO measurements, in a similar way as with the glass vials, disposable borosilicate glass vials with a flat bottom were used, and a sample volume of 8 mL was used (Figure 1). Borosilicate glass vials were preferred to glass vials because of their flat bottom and thinner walls. Measuring from the base (Figure 1a) of the disposable borosilicate glass vials resulted in a slightly higher repeatability of the recorded spectra since when measuring from the nozzle (Figure 1b) of the vials, very slight deviations from the orthogonal configuration between the sample and the instrument resulted in a slight decrease in the signal (the tilting of the instrument causes a noisier average signal), increasing the variability of the recorded spectra. Measurements were then taken from the base by placing the SCiO under the vial (Figure 1a), and replicates were taken by repositioning the vial three times to ensure that the experimental conditions did not change.

#### 3.1.2. NeoSpectra Micro Development Kit

**Figure 2:**
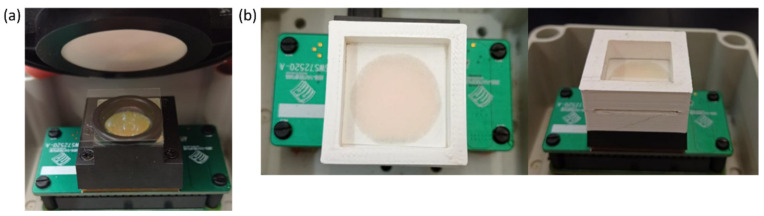
Pictures of the NeoSpectra Micro Development Kit experimental set-ups: (**a**) method 1 (home-made cell) and (**b**) method 2 (paper).

Different measurement strategies were tested, and the device was configured to scan 5 s for each reading. Higher measuring times may affect the measurement because of possible changes in the sample’s temperature during the measurement due to the high heating of the NeoSpectra optical window caused by the three tungsten halogen lamps. During the measurements, a background was taken every 60 min. The following two methods were used for the final measurements:

Method 1 (home-made cell): A plastic ring was used to contain the sample, which was glued with Loctite Super Glue 3 to a borosilicate cover slip. A 200 μL (this volume was optimised) drop of the sample was deposited and covered with another borosilicate cover slip on the top of the plastic ring. This volume was enough to cover the whole surface of the optical window included in the plastic ring, ensuring a reproducible optical path in all the measurements. The home-made cell was placed on the top of the NeoSpectra optical window, and the Spectralon reference was placed on the top of the home-made cell (Figure 2a). The transflectance mode was therefore used. From the same sample, three drops were taken on different home-made cells, and five instrumental replicate measurements were taken for each drop. Several tests were conducted to make sure that neither the ring nor the glue did not produce a measurable instrumental signal. Since the three tungsten halogen lamps and the photodetector are placed at the centre of the optical window, information coming from the outer part of the window (where the ring and the glue are located) contributes less to the overall signal, recording mainly the information coming from the central part of the optical window.

Method 2 (paper): A drop of oil of 10 μL (this volume was optimised after several tests with different volumes) was deposited over a 20 × 20 mm manually cut filter paper. The paper was held by the edges, and the drop was deposited in the centre of the paper. We waited 10 min to ensure that the paper absorbed the full volume of the sample. This time is a compromise between the time needed for the paper to fully absorb the oil and the need for rapid analysis to minimise the loss of volatile compounds [19]. The paper was then placed in a holder with a borosilicate cover slip on each side of the paper (Figure 2b). The holder, which was printed with a 3D printer, is specially designed for this function, and it completely covers the optical window of the NeoSpectra, allowing the paper to be placed just over the window. This design allows the paper to always be placed in the same way in the NeoSpectra optical window while preventing the paper from bending and interfering with the passage of light. From the same sample, five different papers were taken, and five instrumental replicate measurements were taken for each paper.

We tried the same strategy but with the Spectralon reflectance standard placed on top of the holder. Since the instrumental signal did not improve, for the sake of simplicity, the decision was to not use the reflectance standard.

A modification of this method consisted of subtracting the signal from the paper: 10 different filter papers were measured, and the mean of these measurements was subtracted to the measurements of oil and paper. The results of this modification did not improve the results without the subtraction of the paper. Therefore, for simplicity, the decision was to not subtract the paper signal.

Apart from these two methods, other measurement strategies were also tested:

- A drop of oil was deposited on a borosilicate cover slip directly above the NeoSpectra, but the information obtained in the spectra was mainly noise.
- The same configuration (drop on a borosilicate cover slip directly above the NeoSpectra) was tried but with an object covered with aluminium foil a few millimetres placed above the drop to enhance the transflectance. This strategy gave a good spectral signal. However, this option was discarded, as the position and shape of the oil droplets were not totally reproducible, greatly affecting the quality of the signal, even trying to optimise different volumes of the droplet (from 25 to 200 μL). The position of the droplet was important since the maximum instrumental signal was obtained for droplets deposited on the centre of the optical window.
- Quartz cuvettes were also tested. Although the goal is developing a cuvette-cleaning free method, we tried using cuvettes to obtain reference spectra with our instruments. Two cuvettes with optical path lengths of 1 mm and 10 mm were used. In both cases, it was necessary to use aluminium foil at the back of the cuvette to enhance the transflectance; otherwise, only noise was obtained. For the 1 mm cuvette, a foil-covered object was used on the back of the cuvette, and the spectral signal obtained was very similar to that obtained with the droplet. As previously pointed out, this method is unfeasible due to the difficulty of cleaning this cuvette and the possible cross-contamination between samples. When using the 10 mm cuvette with aluminium foil at the back side, the spectra were only obtained in the 1350–1700 nm range: for higher wavelengths, the instrumental signal dramatically decreased to practically 0. Because of this, the possibility of using a borosilicate glass vial with a certain volume of olive oil over the optical window of the NeoSpectra was discarded.

### 3.2. Spectroscopic Signals

**Figure 3:**
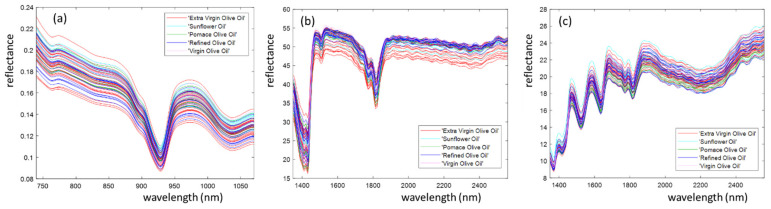
Edible oil spectra recorded using (**a**) SCiO, (**b**) NeoSpectra method 1 (home-made cell) and (**c**) NeoSpectra method 2 (paper).

Figure 3 shows the average reflectance spectra obtained from the analysis of edible oils with SCiO (Figure 3a), NeoSpectra method 1, home-made cell (Figure 3b) and NeoSpectra method 2 with paper (Figure 3c). These data correspond to those produced by the instruments and imported into MATLAB without any pre-treatment. The SCiO and NeoSpectra spectra consisted of 331 and 134 points, respectively. The spectra are coloured according to their commercial classification (extra virgin olive oil, virgin olive oil, refined olive oil, pomace olive oil, sunflower oil). A clear grouping of oils according to their class cannot be seen in either of the instruments and methods tested.

Figure 3a shows two peaks at 930 nm and 1038 nm, which correspond to the vibrations of the methylene group, characteristic of fats and the peak at 980 nm that is related to the water content in oil samples [11]. In Figure 3b,c, the 1390–1490 nm region is related to C–H stretching combinations [12], and the range 1400–1500 nm is related to water absorbance [20], although this range may also include CH_2_ bands from second overtones in olive oil [21]. In the range 1600–1900 nm, there are two peaks representing the vibrations of the methylene group [17], as well as the oleic acid absorbance at 1725 nm [22]. Peaks at 1727 and 1761 nm are attributed to the first overtone of C-H stretching vibrations of methyl, methylene, and ethylene groups [23,24]. The peak in the range 2088–2150 nm corresponds to the fatty acid content [25], with absorbance at 2182 nm corresponding to the NH band absorption [26]. At 2300 nm, there is a peak with a high absorbance corresponding to the combination of fundamental stretching vibrations and other vibrational modes of the CH groups [20,23]. Despite the complementarity of the wavelength ranges used by the two miniaturised instruments, they do not cover the range 1200–1400 nm, which is also important in the analysis of olive oils, including some wavelengths showing the strongest correlation with the colour of several vegetable oils [26].

### 3.3. Multivariate Statistical Analysis

The average of the spectra described in Section 3.1 recorded for each oil sample was used to build the **X** matrix containing information about the instrumental responses. For PLS-DA, a **y** vector was used containing dummy response values related to the two selected classes.

The best spectral pre-processing for SCiO data was found to be second-order polynomial smoothing with 15 points, followed by second Savitzky–Golay derivatives. For NeoSpectra data using method 1 (home-made cell) and method 2 (paper), the best pre-processing was found to be first-order polynomial smoothing with 15 points followed by second Savitzky–Golay derivatives.

#### 3.3.1. Exploratory Data Analysis

**Figure 4:**
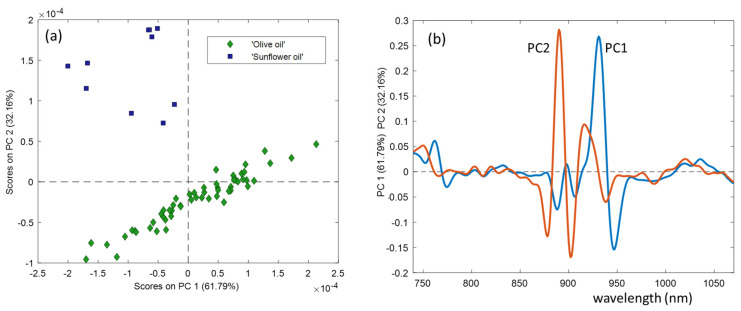
Score (**a**) and loading (**b**) plots for the PCA models between olive and sunflower oils using SCiO data.

A PCA was performed with all the edible oils (all types of olive oils and sunflower oil) and only with all types of olive oils. For both miniaturised devices, the main differentiation between groups of oils in the PCA scores was between sunflower and olive oils. The scores plot for the SCiO data shows a clear difference between olive oils and sunflower oils (Figure 4a). In this plot, the two first PCs explain 93.95% of the information. In the loading plots (Figure 4b), the most important variable in PC1 is 930 nm corresponding to the vibrations of the methylene group, characteristic of fat. The most important variable in PC2, which separates the different types of edible oils, is around 890 nm. For NeoSpectra data, method 1 (home-made cell) is also able to differentiate between olive and sunflower oils, but in this case, looking at the scores plot with the first and fourth PCs.

#### 3.3.2. Classification of Oils

Linear discriminant analysis (LDA) was used to differentiate the samples from the different categories of olive oils, trying to see if the different measurement strategies designed were able to produce good results. The training set used in LDA classification consisted of 38 olive oils, and the test set contained 18 olive oils. These two sets of samples were used in all the measurements and calculations. The three strategies (SCiO, NeoSpectra method 1 and NeoSpectra method 2) were able to correctly predict 100% of the samples in the training set. SciO was able to correctly predict 94% of the samples of the test set, and NeoSpectra method 1 (home-made cell) was able to correctly predict 89% of the samples. The results for NeoSpectra method 2 (paper) were not so good, correctly predicting 50% of the samples. Table 1 shows the sensitivities and specificities for the different categories of olive oils in the training and test set obtained with the application of LDA.

The results in Table 1 show that both SCiO and NeoSpectra are able to provide good classification results. The best results are obtained with SCiO. NeoSpectra using method 1 (home-made cell) also provides good results. NeoSpectra using method 2 (paper) gives poorer predictions of the test set, despite correctly classifying 100% of the samples in the training set. Apart from producing better results, SCiO is easier to use than NeoSpectra when applied to liquid samples such as oil. The results in Table 1 are comparable (especially SCiO results) to the ones reported by different authors classifying different types of olive oils [11,12]. However, it should be mentioned that in these cases, the authors distinguished between extra virgin olive oils and non-extra virgin olive oils but not between different categories of olive oils. Different classification parameters from the ones reported in Table 1, such as precision, accuracy, negative predictive value (NPV), false positive rate (FPR) and false negative rate (FNR) are shown in Table S1 of the Supplementary Materials. The results in Table S1 reinforce the idea that both SCiO and NeoSpectra are able to provide good classification results.

**Figure 5:**
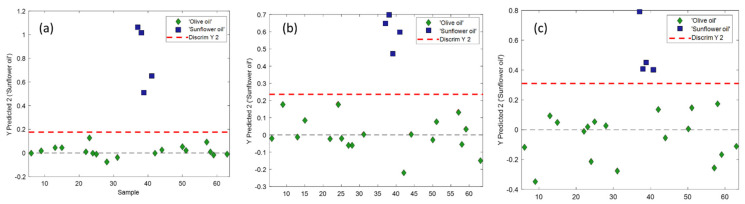
PLS-DA results for the classification of olive and sunflower oils using the test set: (**a**) SCiO, (**b**) NeoSpectra method 1 (home-made cells) and (**c**) NeoSpectra method 2 (paper).

In order to prove the usefulness of the methods of analysis using the two miniaturised instruments, another multivariate classification method, PLS-DA, was used for the differentiation between olive oils (using all the samples in all the classes described previously) and sunflower oil. The same separation of samples used previously in the training and test sets was used for the calculation of the PLS-DA, but in this case, all the different categories of olive oils were removed, and the samples were simply classified as ‘olive oil’. The differentiation between olive and sunflower oils is not trivial from the visual observation of the spectra, as also noted by other authors [12,14,25]; the content of oleic acid is the main difference between both types of oils, with differences also in the contents of palmitic acid, palmitoleic acid, stearic acid and linoleic acid [12]. Figure 5 shows the results of the PLS-DA applied to the different data obtained with the miniaturised instruments. Two latent variables (LVs) were needed in the PLS-DA model with SCiO data to account for 95.49% of the information in **y**. Four LVs were needed in the PLS-DA model with NeoSpectra data (method 1, home-made cell) to account for 89.38% of the information in **y**. Finally, five LVs were needed in the PLS-DA model with NeoSpectra data (method 2, paper) to account for 90.59% of the information in **y**.

Figure 5 shows that all the miniaturised instruments with the different measuring methods designed are able to correctly separate the olive oils from the sunflower oils, with sensitivities and specificities of 1 in all cases.

The three samples of sunflower oil in Figure 5c closer to the discriminant line between the two classes are samples labelled as sunflower oils with a high content of oleic acid. Checking at the labels of olive oils, the monounsaturated fat content exceeds 75%, while in typical sunflower oils, it does not exceed 30%. However, these sunflower oils labelled with high oleic acid content have a percentage of monounsaturated fat around 50%. One can therefore assume that the instruments are also able to correctly classify olive and sunflower oils despite having sunflower oils a high percentage of oleic acid.

Figure S1 in the Supplementary Materials shows the loadings for the three PLS-DA models. Figure S1a shows that the most important variable for the classification model in SCiO is 930 nm corresponding to the vibrations of the methylene group, characteristic of fats. In Figure S1b, corresponding to NeoSpectra method 1 (home-made cells), the most important parts correspond to the range of 1400–1500, water absorbance and CH_2_ bands from second overtones in olive oil and the range 1700–1900, which, among others, includes the information for oleic acid. Loadings in Figure S1c, NeoSpectra method 2 (paper) are more difficult to interpret because the information in **y** is not concentrated on the two first LVs.

It is worth mentioning that, initially, in the NeoSpectra Micro Development Kit optimisation process, a reduced number of olive and sunflower oils (not the whole set of samples) were analysed, placing an 80 μL droplet on a borosilicate cover slip directly above the NeoSpectra and placing an object covered with an aluminium foil a few millimetres above the drop (transflectance measurements). Although, as mentioned above, this strategy was discarded because the position and the shape of the oil droplet were not totally reproducible (the shape and the optical path was also affected by the viscosity of the individual oils), a PLS-DA with cross-validation was able to differentiate between olive and sunflower oils in this reduced set of samples.

## 4. Conclusions

This manuscript describes the design and optimisation of the measurement methods of two miniaturised low-cost NIR instruments applied to the analysis and classification of commercial olive oils. Being a challenging sample to be measured, especially using NeoSpectra, as shown from the measuring strategies developed, the different tested strategies provide good results when applied to the classification of olive oils without any sample pre-treatment. Two different methods of classification have been applied that successfully discriminate the different categories of olive oils and olive oil from sunflower oil. Good results of the different parameters used to assess the goodness of the classification were obtained using the LDA classification technique (especially using the SCiO results), and PLS-DA was successfully used in the differentiation of olive and sunflower oil. The results suggested that both devices have a potential to be used, e.g., in quality control phases in the olive oil industry.

Miniaturised NIR instruments are rapidly expanding, and they are increasingly being applied to a wide variety of samples in many different environments and situations. Nevertheless, the best operational conditions must be optimised to assure the best quality signal and, therefore, to obtain the best results, as shown in the paper. This may involve intensive work optimising and developing suitable measurement conditions involving the specific properties of the samples to be measured and the specific characteristics of the instruments to use. This optimisation process may require extensive experience in analytical measurement and also in the use of chemometric methods.

## Figures and Tables

**Table 1:** Classification of different classes of olive oils applying LDA classification to SCiO and NeoSpectra data.

		Training Set Sensitivity	Training Set Specificity	Test Set Sensitivity	Test Set Specificity
SCiO	Extra virgin olive oil	1	1	1	0.88
	Pomace olive oil	1	1	1	1
	Refined olive oil	1	1	0.80	1
	Virgin olive oil	1	1	1	1
NeoSpectra method 1 (home-made cells)	Extra virgin olive oil	1	1	1	0.88
	Pomace olive oil	1	1	1	1
	Refined olive oil	1	1	0.60	1
	Virgin olive oil	1	1	1	0.94
NeoSpectra method 2 (paper)	Extra virgin olive oil	1	1	0.60	0.63
	Pomace olive oil	1	1	0	1
	Refined olive oil	1	1	0.40	0.85
	Virgin olive oil	1	1	1	0.76

## Supplementary Materials

The following are available online at https://www.mdpi.com/article/10.3390/foods10112856/s1, Table S1: Classification of different classes of olive oils applying LDA classification to SCiO and NeoSpectra data. Figure S1. PLS-DA loadings for (a) SCiO, (b) NeoSpectra method 1 (home-made cells), (c) NeoSpectra method 2 (paper).
