# Supplementary material for: Measurement Strategies for the Classification of Edible Oils Using Low-Cost Miniaturised Portable NIR Instruments
Source: Foods. 2021 Nov 18;10(11):2856. doi: 10.3390/foods10112856 (PMC8618161; doi:10.3390/foods10112856)
Supplement: Supplementary file 1 [file foods-10-02856-s001.zip › foods-1449036-supplementary.pdf]

Measurement strategies for the classification of edible oils using low-cost miniaturised portable NIR instruments

Barbara Giussani, Alix Tatiana Escalante-Quiceno, Ricard Boqué and Jordi Riu

Supplementary Materials

Table S1. Classification of different classes of olive oils applying LDA classification to SCiO and NeoSpectra data.

		Precision	Accuracy	NPV	FPR	FNR
**SCiO**	**EVOO training set**	1	1	1	0	0
	**POO training set**	1	1	1	0	0
	**ROO training set**	1	1	1	0	0
	**VOO training set**	1	1	1	0	0
	**EVOO test set**	0.91	0.94	1	0.13	0
	**POO test set**	1	1	1	0	0
	**ROO test set**	1	0.94	0.93	0	0.20
	**VOO test set**	1	1	1	0	0
**NeoSpectra method 1 (home-made cells)**	**EVOO training set**	1	1	1	0	0
	**POO training set**	1	1	1	0	0
	**ROO training set**	1	1	1	0	0
	**VOO training set**	1	1	1	0	0
	**EVOO test set**	0.91	0.94	1	0.13	0
	**POO test set**	1	1	1	0	0
	**ROO test set**	1	0.89	0.87	0	0.40
	**VOO test set**	0.50	0.94	1	0.06	0
**NeoSpectra method 2 (paper)**	**EVOO training set**	1	1	1	0	0
	**POO training set**	1	1	1	0	0
	**ROO training set**	1	1	1	0	0
	**VOO training set**	1	1	1	0	0
	**EVOO test set**	0.67	0.61	0.56	0.38	0.40
	**POO test set**	-	0.89	0.89	0.00	1.00
	**ROO test set**	0.50	0.72	0.79	0.15	0.60
	**VOO test set**	0.20	0.78	1	0.24	0

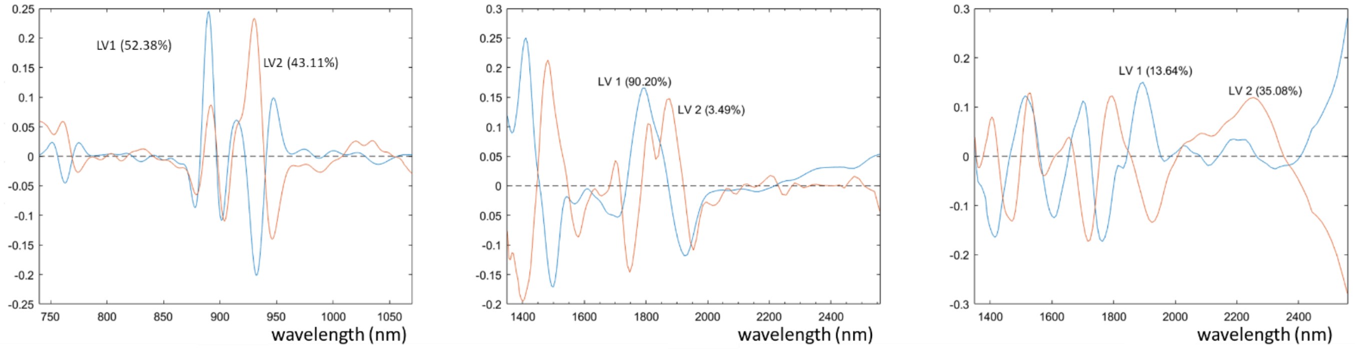

Figure S1. PLS-DA loadings for a) SCiO, b) NeoSpectra method 1 (home-made cells), c) NeoSpectra method 2 (paper).

For the sake of clarity, in the case of NeoSpectra only the loadings for the 2 first latent variables are shown in Figure S1.
